# Health related quality of life of HIV-positive women on ART follow-up in north Shewa zone public hospitals, central Ethiopia: Evidence from a cross-sectional study

**DOI:** 10.1016/j.heliyon.2023.e13318

**Published:** 2023-01-29

**Authors:** Derara Girma, Hiwot Dejene, Leta Adugna Geleta, Mengistu Tesema, Elsabeth Legesse, Tadesse Nigussie, Berhanu Senbeta Deriba, Tinsae Abeya Geleta, Degemu Sahlu, Addisu Waleligne, Sisay Abebe Debela, Mukemil Awol

**Affiliations:** a Department of Public Health, College of Health Science, Salale University, Ethiopia; b Department of Midwifery, College of Health Science, Salale University, Ethiopia

**Keywords:** HIV/AIDS, Antiretroviral therapy, Quality of life, Women, Ethiopia

## Abstract

**Background:**

Evidence revealed that there is a statistically significant gender difference in Health-related quality of life (HRQoL) among HIV-positive people on Antiretroviral therapy (ART). Consequently, HIV-positive women have low scores in all HRQoL domains than men. Despite this fact, previous studies in Ethiopia focused on general HIV-positive people and paid less attention to HIV-positive women. Therefore, this study was intended to measure HRQoL and associated factors among HIV-positive women on ART follow-up in north Shewa zone public hospitals, central Ethiopia.

**Methods:**

An institution-based cross-sectional study was conducted from February 01-April 30, 2022. Four hundred twenty-six women on ART were included using a systematic random sampling technique. Face-to-face interviews and medical record reviews were used to collect data. Both bivariable and multiple linear regressions were computed to identify the factors associated with HRQoL. A p-value <0.05 was used to assert statistically significant variables in multiple linear regression analysis.

**Results:**

The overall mean (SD) score of the HRQoL was 11.84 (2.44). And, 44.7% [95% CI: 40.3, 49.5] of the women have poor HRQoL. In multiple linear regression analysis, factors like depression (β = −0.35), Post-Traumatic Stress Disorder (PTSD) (β = −0.16), age (β = −0.07), rural residence (β = −0.52), and bedridden functional status (β = −1.02) were inversely associated with HRQoL. Oppositely, good treatment adherence (β = 0.46) was positively associated with overall HRQoL, keeping other factors constant.

**Conclusion:**

This study reveals a high magnitude of poor HRQoL among HIV-positive women. Therefore, as HRQoL domains are comprehensive indicators of living status, healthcare service providers should be dedicated to screening and supporting HIV-positive women with poor HRQoL. Additionally, healthcare providers should also pay special attention to routine PTSD screening and management for HIV-positive women due to its detrimental effect on HRQoL.

## 1 Introduction

Human Immuno-deficiency Virus/Acquired Immuno-deficiency Syndrome (HIV/AIDS) remains a major global public health concern, infecting 84.2 million [64.0 million-113.0 million] people and killing 40.1 million [33.6–48.6 million] people to date [1]. By the end of 2021, there were an estimated 38.4 million [33.9–43.8 million] people living with HIV [1] of which 54% were women and girls [2]. In the same year, 1.5 million [1.1 million-2.0 million] people became newly infected with HIV, with women and girls accounting for 49% [2]. Concerning the regional burden, African women and girls are disproportionately affected by HIV/AIDS, accounting for 63% of HIV patients and 60% of new infections in Africa [3]. Also in Ethiopia, women account for 59% of HIV patients and 51% of new infections [4].

Quality of Life (QoL) is an individual's perception of their position in life in the context of the culture and value systems in which they live and about their goals, expectations, standards, and concerns. QoL is also used as one of the widely accepted theoretical frameworks for assessing the living conditions of patients and it also is recognized as a key component of public health [5]. HRQoL is a multidimensional and complex concept and reflects subjective perceptions of individuals, their physical health, psychological state, level of independence, social relationships, personal beliefs, and relationship to salient features of their environment [6,7]. HRQoL is referring to the impact of disease and treatment on QoL. It is a core concept that comprises mostly self-reported measures of physical and mental health dimensions, and it has become an increasingly popular subjective health evaluation method in chronic diseases [8].

HIV/AIDS is often excluded from the list of chronic diseases though it is widely recognized as a chronic illness within HIV care [9]. Subsequently, HIV-positive people have significantly lower HRQoL than the general population, even among virologically and immunologically stable patients [10]. Because, AIDS impairs HRQoL, which is an important measurable outcome of HIV treatment in the era of Highly Active Antiretroviral Treatment (HAART), complementing more objective outcomes such as death [11].

Evidence revealed that there is a statistically significant gender difference in HRQoL among HIV-positive people on HAART; women have low scores in all HRQoL domains as compared to men [[12], [13], [14]]. On top of the gender difference, studies also indicated that HIV-positive women have significantly poorer functioning and well-being than HIV-negative women [15]. As a result, there is now mounting evidence that constitutes HRQoL as an important area of research and there is a need for further investigation along with the factors that influence it among HIV-positive women [16].

As evinced so far, factors like frequent HIV symptoms [17], anxiety [18], depression [13,[17], [18], [19], [20]], perceived stigma [12,17,21], food insecurity [19], TB/HIV co-infection [13], rural residence [13], and being anemic [13] were negatively associated with HRQoL in HIV-positive women. Oppositely, factors like problem-focused coping [17], perceived situational control [17], healthy lifestyles [17], social support [13,18,21,22], higher wealth status [22], higher CD4 count [21], and resilience [19] were positively associated with HRQoL in the similar population. Despite this documentation, the stipulation for more studies to measure variation in HRQoL over time is suggested [23].

Even though there is a gender difference in HRQoL, previous studies in Ethiopia focused on general HIV-positive people and paid less attention to HIV-positive women [[24], [25], [26]]. Aside from the scarcity of studies on HRQoL among HIV-positive women in Ethiopia, there was no similar study in the specific study area. Therefore, this study was intended to measure HRQoL and associated factors among HIV-positive women on ART follow-up in north Shewa zone public hospitals, central Ethiopia.

## 2 Methods

### 2.1 Study area, design, and period

This study was conducted in the North Shewa zone, Oromia regional state, central Ethiopia. Four public hospitals that are currently providing HIV/AIDS care and treatment (namely: Salale university comprehensive specialized hospital, Kuyu General Hospital, Sheno health center, and Gundo-meskel primary hospital) were included in the study. A cross-sectional study was conducted from February 01-April 30, 2022 among HIV-positive women (aged 18 and above years) attending ART clinics in public hospitals of the zone.

### 2.2 Sampling procedures

A single population mean formula was used to calculate the sample size. Accordingly, from a previous study [22] standard deviation (σ) = 2.19448 and n = 344 were considered. Likewise, a 95% confidence level, and 15% contingency were applied. Thus, from the above parameters

nf = ((Z_α/2_)^2^ *σ^2^) / d^2^

n_f_ = ((1.96)^2^ * (2.19448)^2^) / (0.23128)^2^

Nf = 370. Thus, after a 15% contingency was added, 426 women were sampled. Then, the sample size was distributed using proportional allocation to size (PAS) to each ART clinic of the included public hospitals. Finally, a systematic random sampling technique was used to recruit the study participants.

### 2.3 Measurements

HRQoL was measured using the WHO QoL-HIV BREF scale among HIV/AIDS patients. The questionnaire contains 31 items distributed into 6 domains: physical, social relationships, level of independence, and spirituality domains each with 4 items, and psychological and environmental domains with 5 and 8 items, respectively. The individual items are rated on a 5-point Likert scale where 1 indicates low/negative perceptions and 5 indicates high/positive perceptions. The remaining two items measure the overall perceived quality of life and general health perception of women living with HIV [27]. The tool was validated in Ethiopian HIV-positive women (Cronbach's α > 0.80). As well, the scale's six domains exhibited good concurrent validity, with a coefficient of r = 0.63–0.82 [28]. Furthermore, the WHO QoL-HIV BREF scale tool demonstrated excellent reliability among HIV-positive women in Ethiopia (Cronbach-alpha = 0.89) [22]. The tool was also reliable in this study (Cronbach α = 0.92).

Anxiety and Depression were measured by Hospital Anxiety and Depression Scale (HADS). The tool is validated in Ethiopian HIV-positive patients. The internal consistency of the HADS was 0.78, 0.76, and 0.87 for the anxiety and depression subscales and the full scale, respectively. The intra-class correlation coefficient (ICC) was 80%, 86%, and 84% for the anxiety and depression subscales, and total scores respectively [29]. The tool was also reliable in this study (Cronbach α = 0.72).

The wealth index was measured using 15 simplified household assets questions available from www.equitytool.org. Accordingly, the wealth index of the household was classified into five quintiles. Then, the wealth index of the 1st and 2nd quintiles were classified as poorest, those in the 3rd quintile were middle, and those in the 4th and 5th quintiles were richest. The tool has 84.2% agreement and 0.76 kappa statistics with the 2016 Ethiopian Demographic Health Survey (EDHS) wealth index questionnaire [30].

Social support was measured by Oslo‐3 Social Support Scale (OSS‐3). The tool comprises valid values ranging from 3 to 14. A score ranging from 3 to 8 is classified as “poor support”, 9–11 is classified as “intermediate support”, and 12–14 is classified as “strong support”. The tool has a Cronbach-alpha of 0.88 [31]. The tool was also reliable in this study (Cronbach α = 0.73).

Perceived stigma was assessed by a 10-item perceived HIV stigma scale. The level of perceived stigma was measured by Likert scale questions (strongly disagree - strongly agree) with a value of 1–5, respectively. The study participants who scored mean and above from 10 stigma assessment questions were classified as having perceived stigma [32]. The tool was also reliable in this study (Cronbach α = 0.73).

Post-Traumatic Stress Disorder (PTSD) was measured by the Primary Care PTSD Screen for DSM-5 (*P*C-PTSD-5) tool. The tool has five items with binary options (yes/no) and a score ≥3 indicates the presence of PTSD symptoms [33]. The tool was also reliable in this study (Cronbach α = 0.76).

Adherence to ART was measured based on patients’ recall of their compliance with the prescribed doses in the last 30 days. Patients who reported an intake of ≥95% of the prescribed medication were considered good adherent, and those with a reported intake of <95% were classified as poor adherent [34].

### 2.4 Data collection procedures

Data were collected through a face-to-face interview and a review of medical records based on the proportion of HIV-positive women allocated to each hospital. Specifically, data on the women's socio-demographic, clinical, and psycho-social characteristics were collected using pretested semi-structured interview-administered questionnaires. In addition, data on the women's HIV-related characteristics were extracted from medical records. Four trained nurses collected data under the supervision of two public health professionals.

### 2.5 Statistical analysis

The collected data were entered into Epi data version 3.1 and then exported to SPSS version 25 for analysis. Participant characteristics were described using descriptive statistics like frequency and percentage, mean and standard deviation (SD), and median and inter-quantile range (IQR). Only variables significant at p-value <0.25 in simple linear regression were included in subsequent multiple linear regression analysis. In multiple linear regression, variables with a p-value <0.05 were taken as statistically significant. Results were presented as β-coefficient with a 95% confidence interval (CI), and p-value. The assumptions of least squares regression (linearity, normal distribution, equal variances, and independent observations) were checked. Thus, assumptions of linearity and equal variances were checked by visual inspection of scatter plots, and there was no clear pattern on the scatter plot. The normal probability plot showed that the error term was normally distributed. The low variance inflation factor (<2.8) indicated that the associated independent variable has lower collinearity with the other variables in the model. A good fit model for multiple linear regression was determined (Adjusted R^2^ = 0.737).

### 2.6 Data quality assurance

WHO QoL-HIV BREF scale is a validated and well-adapted tool for Ethiopian HIV-positive women [28]. Besides, all questionnaires were translated into the local languages (Afan Oromo and Amharic) by two independent bilingual translators and back-translated to English to guarantee consistency. A one-day training was given to the data collectors and supervisors. Ten percent (10%) of the questionnaire was pre-tested at Chancho general hospital and mandatory clarifications and modifications on ambiguous points were made. Likewise, a reliability test was done and tools with Cronbach-alpha >0.7 were used during the actual data collection.

## Ethical approval

An ethical approval letter was obtained from the Institution review board of Salale University. A permission letter was secured from each health facility's administration. The purpose of the study was informed to the patients and written informed consent was obtained from each participant before the interview. Additionally, all the information obtained from each study participant was kept confidential throughout the process of this study.

## 3 Results

### 3.1 Sociodemographic characteristics

The response rate of the study was 97%. The women's mean (SD) age was 36 [9] years old. And, the age range was 47 (minimum 18 and maximum 65) years old. About 277 (67.2%) of the women were urban residents and more than half, 217 (52.7%) were married followed by widowed, 100 (24.3%). Moreover, about 19 (28.9%) of the women lack formal education and about 160 (38.8%) were in the poorest wealth index category (Table 1).

**Table 1:** Socio-demographic characteristics of the HIV-positive women attending ART clinics in north Shewa zone public hospitals (n = 412).

Variable name	Category	Freq. (%)
Age	18–28	97 (23.5)
	29–39	177 (43.0)
	40^+^	138 (33.5)
Residence	Rural	135 (32.8)
	Urban	277 (67.2)
Marital status	Divorced	41 (10.0)
	Married	217 (52.7)
	Single	54 (13.1)
	Widowed	100 (24.3)
Educational status	No formal education	119 (28.9)
	primary (1–8 grade)	96 (23.3)
	Secondary (9–12)	78 (18.9)
	College and above	119 (28.9)
Wealth index category	Poorest (1st and 2nd quintiles)	160 (38.8)
	Middle (3rd quintile)	88 (21.4)
	Richest (4th and 5th quintiles)	164 (39.8)
Occupation status	Housewife	159 (38.6)
	Government employee	92 (22.3)
	Private employee	44 (10.7)
	Student	35 (8.5)
	Unemployed	78 (18.9)
	Others^a^	4 (1.0)

a Daily laborer, driver.

### 3.2 Clinical characteristics

Of the total women, 364 (88.3%) have been above 60 months since diagnosed with HIV/AIDS. The mean (SD) baseline CD4 was 200 (52), while the mean (SD) current CD4 was 500 (100). Additionally, 305 (74.0%) were in baseline stage 3 WHO clinical stage, while 300 (72.8%) were currently in WHO clinical stage 1. Moreover, 230 (55.8%) of the participants had one form of opportunistic infection. Tuberculosis, 79 (19.2%) was the top opportunistic infection. Besides, 68 (16.5%) of the participants were non-adherent to the treatment (Table 2).

**Table 2:** Clinical characteristics of the HIV-positive women attending ART clinics in north Shewa zone public hospitals (n = 412).

Variable	Category	Freq. (Percentage)
Duration since diagnosed	less than 60 months	48 (11.7)
	above 60 months	364 (88.3)
Treatment adherence	Adherent	344 (83.5)
	Non-adherent	68 (16.5)
Baseline WHO clinical stage	Stage 1	14 (3.4)
	Stage 2	57 (13.8)
	Stage 3	305 (74.0)
	Stage 4	36 (8.7)
Current WHO clinical stage	Stage 1	300 (72.8)
	Stage 2	86 (20.9)
	Stage 3	8 (1.9)
	Stage 4	18 (4.4)
Opportunistic infections	Yes	230 (55.8)
	No	182 (44.2)
Current ART regimen	1st line	286 (69.4)
	2nd regimen	118 (28.6)
	3rd regimen	8 (1.9)
ARV side effects	Yes	113 (27.4)
	No	299 (72.6)
Current functional status	Working	312 (75.7)
	Bedridden	12 (2.9)
	Ambulatory	88 (21.4)

### 3.3 Psychosocial and behavioral characteristics

Of the total women, 21.1% and 35.2% have anxiety and depression respectively. Additionally, 75.7% of women have poor social support. Furthermore, 49.8% of the women reported suffering from perceived stigma, while 27.7% were screened positive for Post-Traumatic Stress Disorder (PTSD). About 19% of the women stated that they are currently using substances (either alcohol, cigarette, or shisha) (Table 3).

**Table 3:** Psychosocial and behavioral characteristics of HIV-positive women attending ART clinics in north Shewa zone public hospitals (n = 412).

Variable name	Category	Freq. (Percentage)
Anxiety	Present	87 (21.1)
	Absent	325 (78.9)
Depression	Depressed	145 (35.2)
	Non-depressed	267 (64.8)
Social support	Poor	312 (75.7)
	Intermediate	96 (23.3)
	Strong	4 (1.0)
Perceived stigma	Stigmatized	205 (49.8)
	Not-stigmatized	207 (50.2)
Post-Traumatic Stress Disorder	Screened positive	114 (27.7)
	Screened negative	298 (72.3)
Current substance use	Yes	78 (18.9)
	No	334 (81.1)

### 3.4 Health-related quality of life

The overall mean (SD) of the HRQoL was 11.84 (2.44) (Table 4). And, 44.7% [95% CI: 40.3, 49.5] of the women have poor HRQoL. Furthermore, on perceived measures of general QoL and health status, 53.4% of participants reported good general QoL, while 11.6% reported poor/very poor. In terms of perceived satisfaction with their health, 25.5% of participants were dissatisfied/very dissatisfied, while 60.9% were satisfied/very satisfied.

**Table 4:** Overall and domains’ HRQoL mean score of HIV-positive women attending ART clinics in north Shewa zone public hospitals (n = 412).

HRQoL domains	Items	Mean	Standard deviation	Minimum	Maximum
Physical domain	4	10.66	2.77	4	18
Psychological domain	5	12.44	3.04	4	17.6
Level of independence	4	11.74	2.43	6	19
Social relationship	4	13.20	3.24	4	20
Environmental	8	12.67	2.82	5.50	17.50
Spirituality	4	10.31	2.81	5	20
Overall HRQoL	29	11.84	2.44	5.58	16.27

#### 3.4.1 Domain of HRQoL

**Fig. 1:**
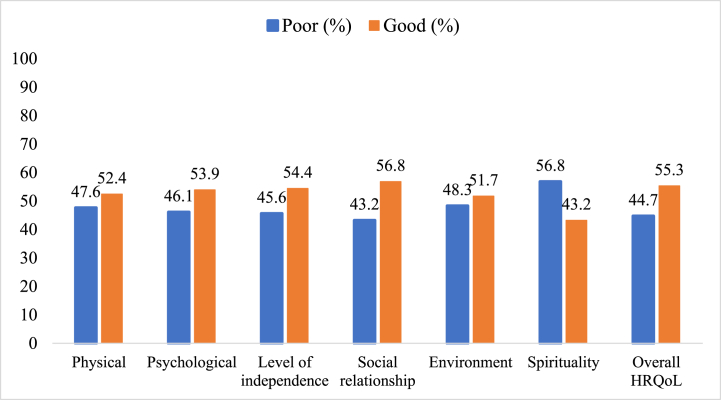
Total and each domain HRQoL status of HIV-positive women attending ART clinics in north Shewa zone public hospitals (n = 412).

Among the HRQoL domains, the spirituality domain had the highest rate of poor HRQoL (56.8%) followed by the environment domain (48.3%). In contrast, the social relationship was found to have the minimum poor HRQoL (43.2%) (Fig. 1).

#### 3.4.2 Factors associated with HRQoL

In simple linear regression analysis factors like depression, Post-Traumatic Stress Disorder, age of the women, being rural residents, current WHO clinical stage-2, duration since HIV diagnosis, bedridden functional status, comorbidity of chronic disease(s), and current substance use were inversely associated with a higher score of HRQoL. However, good treatment adherence, strong social support, and a higher current CD4 count were factors directly associated with a higher score of HRQoL (Table 5).

**Table 5:** Simple and multiple linear regression for factors associated with HRQoL among HIV-positive women attending ART clinics in north Shewa zone public hospitals (n = 412).

Variable	Simple linear regression		Multiple linear regression	
	Unstandardized coefficients (β)	95% CI for β	Unstandardized coefficients (β)	95% CI for β
Age	**−0.18**	**(-0.19, -0.16)**	**−0.07**	**(-0.09, -0.05)**^a^
Rural residence	**−1.73**	**(-2.21, -1.26)**	**−0.52**	**(-0.79, -0.24)**^a^
Good treatment adherence	**0.85**	**(0.22, 1.48)**	**0.46**	**(0.12, 0.79)**^a^
Depression	**−0.52**	**(-0.55, -0.48)**	**−0.35**	**(-0.40, -0.31)**^a^
Strong social support	1.45	(-0.96, 3.86)	−0.28	(-1.59, 1.02)
Current CD4 count	0.10	(0.008, 0.12)	0.001	(-0.0003, 0.003)
Current WHO clinical stage-2	−2.81	(-3.32, −2.29)	−0.33	(0.68, 0.03)
Duration since HIV diagnosis	−0.018	(-0.022, −0.015)	−0.001	(0.004, 0.002)
Bedridden functional status	**−5.77**	**(-7.07, -4.48)**	**−1.02**	**(-1.84, -0.21)**^b^
Comorbidity of chronic disease(s)	−0.75	(-1.47, −0.03)	0.09	(-0.29, 0.48)
Post-Traumatic Stress Disorder	**−0.49**	**(-0.70, -0.29)**	**−0.16**	**(-0.28, -0.04)**^b^
Current substance use	−1.97	(-2.55, −1.40)	0.29	(-0.07, 0.65)

a Significant at p-value <0.001.

b Significant at p-value <0.05.

In multiple linear regression analysis, factors like depression, Post-Traumatic Stress Disorder, age of the women, rural residents, and bedridden functional status were inversely associated with a higher score of HRQoL. On the other hand, good treatment adherence was positively associated with an overall higher score of HRQoL, keeping other factors constant (Table 5).

Accordingly, as women's age increase by one year, their HRQoL score is expected to fall by 0.07 unit averagely (β = −0.07). Rural women were supposed to have a 0.52 lower HRQoL score than urban women (β = −0.52). Besides, as the depression scale increases by a unit, the women's HRQoL score is reduced by 0.35 units (β = −0.35). The rate of the average change in HRQoL among women who are bedridden functional status is 1.02 lower compared to women who are ambulatory/working functional status (β = −1.02). Moreover, as the PTSD scale increase by one unit, the HRQoL is anticipated to decrease by 0.16 units (β = −0.16). In contrast to the above, women who have good treatment adherence are expected to have a 0.46 higher HRQoL score as compared to women who have poor treatment adherence status (β = 0.46) (Table 5).

## 4 Discussion

This study measures the HRQoL and associated factors among HIV-positive women. The overall mean (SD) of the HRQoL was 11.84 (2.44) and 44.7% of the women have poor HRQoL. This finding was lower than the study conducted among HIV-positive women in Jimma, Ethiopia (53.5%) [22]. HRQoL might increase as a result of time changes, service expansion, and improvements. In contrast, the level of poor HRQoL in this study was higher than the study from south India (7.9%) [35]. This could be attributed to differences in the status of the enrolled women, the sample size, and socio-demographic factors.

However, the level of poor HRQoL in this study was found to be consistent with the pooled prevalence of HRQoL among general HIV-positive people in Ethiopia (45.27%) [36]. Over time monitoring of HRQoL will support HIV care and treatment practices to settle auspicious outcomes [23]. Additionally, the study from Indonesia reported a comparable finding (45%) [37]. The similarity in the use of the WHO QoL-HIV BREF tool to collect data might result in equivalent findings.

In this study, age was inversely associated with HRQoL. Studies from India [38] and Canada [19] have reported comparable findings. This might be due to the complex interactions of health-related socio-ecological factors affecting aging among HIV-positive women [19]. Conversely, the studies from Northeast Ethiopia [39] and the southeastern United States [40] reported opposing findings. Having a stable home environment and fewer home responsibilities, and perceiving fewer wasted chances in furthering career goals among older women may lead to better HRQoL [40]. A place of residence was another socio-demographic factor associated with HRQoL. Consequently, rural HIV-positive women had lower HRQoL scores. The previous studies conducted in western [13] and northern [12] Ethiopia confirmed this. In rural residents, the presence of relatively poor infrastructure, greater financial inadequacy, high stigmatization, and marginalization may result in poor HRQoL.

Being well adhered to ART was found to increase HRQoL score. The previous literature review and a study from China found that ART adherence improves HRQoL [41,42]. As well, a study from Colombia found that non-adherence to combined ART was associated with lower QoL [43]. Improving patients’ devotion and compliance toward their treatment, health, and understanding of the significance of ART adherence is crucial. In contrast, a study of HIV-positive transgender women in Sao Paulo, Brazil, found no association between these two variables [44].

In the assessment of the mental health domain, depression was found to have a negative association with HRQoL. A study from Thailand also confirms this finding [20]. Additionally, a considerable body of studies shows that depression has a significant negative effect on QoL among HIV-positive people [17,19,[44], [45], [46]]. This suggests the necessity to reconsider mental health care strategies for HIV-positive women. Moreover, the presence of PTSD symptoms was also inversely associated with HRQoL. Comparably, previously reported that the occurrence of PTSD symptoms is threatening HIV-positive people QoL [47]. This is because HIV is one of the trauma-inducing chronic illnesses with negative effects on HRQoL. Another study also implied that poor adherence to antiretroviral therapy is a pathway through which PTSD symptoms exert a negative influence on the HRQoL of HIV-positive people [48]. Consequently, early detection of PTSD among HIV-positive people is crucial to address the issue [49].

### 4.1 Strengths of the study

In this study, a locally validated HRQoL measuring tool was used, and the tool's reliability was also comprehensively analyzed in this study. Also, the current findings have implications for medical/public health decision-making in terms of designing evidence-based interventions considering the chronic nature of HIV infection and patient-disclosed outcomes on HRQoL. Moreover, the identification of psychological risk factors like PTSD as being associated with poor HRQoL provides a window of opportunity to improve HRQoL; thus, intervention strategies to prevent and control PTSD should be viewed as critical to HIV-positive women.

### 4.2 Limitations of the study

The cross-sectional nature of the study does not allow temporality ascertainment. Furthermore, recall bias and social desirability biases may exist because participants were asked to respond based on their life experiences. Additionally, HIV-positive women who were unaware of their sero-status, were not receiving care, or were hospitalized did not participate in this study — the findings cannot be generalized to these populations.

## 5 Conclusion

This study found that nearly half of the HIV-positive women have poor HRQoL. Moreover, the age of the women, rural residents, depression, bedridden functional status, and PTSD were inversely associated with a higher score of HRQoL. Conversely, good treatment adherence was positively associated with an overall higher score of HRQoL. Subsequently, because HRQoL domains are comprehensive indicators of living status, HIV-positive women with poor HRQoL should be identified and supported. Furthermore, interventions aimed at improving HRQoL in HIV-positive women should incorporate/reinforce approaches for routine PTSD and depressive symptoms screening and management due to their negative impact on HRQoL. Moreover, routine assessment of treatment adherence and apt interventions at every clinic visit should be encouraged to increase the HRQoL of HIV-positive women. Also, HIV-positive women who are elderly, bedridden, and live in rural areas should be given special consideration due to their potential poor HRQoL. More research on similar populations using other HRQoL measuring tools than the WHO-QOL-HIV BREF is also suggested to address the issue of tool invariance and thus facilitate comparability. Besides, future research should attempt to identify novel variables that influence the level of HRQoL in diverse contexts.

## Declarations

### Author contribution statement

Derara Girma, Hiwot Dejene, and Leta Adugna Geleta: Conceived and designed the experiments; Performed the experiments; Analyzed and interpreted the data; Contributed reagents, materials, analysis tools or data; Wrote the paper.

Mengistu Tesema, Elsabeth Legesse, Tadesse Nigussie, Berhanu Senbeta Deriba, Tinsae Abeya Geleta, Degemu Sahlu, Addisu Waleligne, Sisay Abebe Debela, and Mukemil Awol: Performed the experiments; Contributed reagents, materials, analysis tools or data; Wrote the paper.

### Funding statement

This research work was funded by 10.13039/501100023324Salale University, Ethiopia [Ref. No.: SlU-IRB-023-2022]

### Data availability statement

Data will be made available on request.

### Declaration of interest's statement

The authors declare no competing interests.
