# Supplementary material for: Health related quality of life of HIV-positive women on ART follow-up in north Shewa zone public hospitals, central Ethiopia: Evidence from a cross-sectional study
Source: Heliyon. 2023 Jan 29;9(2):e13318. doi: 10.1016/j.heliyon.2023.e13318 (PMC9898445; doi:10.1016/j.heliyon.2023.e13318)
Supplement: Multimedia component 2 [file mmc2.docx]

**English version questionnaire**

| **Part One: Questions related to sociodemographic characteristics** | | | |
| --- | --- | --- | --- |
| S/N | Questions | Possible responses | skip |
| 101 | how old are you? | _________ (in a completed year) |  |
| 102 | where do you live? | 1. Urban 2. Rural |  |
| 103 | what is your current marital status? | 1. Single 2. Married 3. Divorced   4. Widowed 5. others (Specify)________ |  |
| 104 | what is your religion? | 1. Muslim 2. Orthodox 3. Protestant   4. Catholic 5. (Other) specify __________ |  |
| 105 | what is your educational status? | 1. No formal education 2. Read and write   3. Primary 4. Secondary 5. College & above |  |
| 106 | What is your occupation? | 1. Unemployed 2. Government employee   3.Merchant 4. Student 5. Farmer   1. (Other) specify __________ |  |
| **Part Two: Questions related to household wealth index** | | | |
| 201 | Does your household have electricity? | 1. Yes 2. No |  |
| 202 | Does your household have a radio? | 1. Yes 2. No |  |
| 203 | Does your household have a television? | 1. Yes 2. No |  |
| 204 | Does your household have refrigerator? | 1. Yes 2. No |  |
| 205 | Does your household have electric ‘mitad’? | 1. Yes 2. No |  |
| 206 | Does your household have a table? | 1. Yes 2. No |  |
| 207 | Does your household have a chair? | 1. Yes 2. No |  |
| 208 | Does your household have a bed with cotton sponge/spring mattress? | 1. Yes 2. No |  |
| 209 | Does any member of this household have a bank account? | 1. Yes 2. No |  |
| 210 | What is the main source of drinking water? | 1. Piped to yard/plot 2. Other |  |
| 211 | What kind of toilet facility do members of your households usually use? | 1. Pit latrine without slab/open pit 2. No facility/bush/open 3. Other |  |
| 212 | What types of fuel does your household use for cooking? | 1. Electricity 2. Wood 3. Other |  |
| 213 | What is the main material of the floor in your household? | 1. Earth 2. Other |  |
| 214 | What is the main material of the exterior walls in your household? | 1. Bamboo with mud 2. Other |  |
| 215 | What is the main material of the roof in your household? | 1. Corrugated iron/metal 2. Other |  |
| **Part Three: Clinical factors related questions** | | | |
| 301 | Have you missed the ART dose over the last 2 weeks | 1. Yes 2. No | if “no” go-to Q303 |
| 302 | Number of doses missed over the last 2 weeks | ________ (in number) |  |
| 303 | Number of doses has to be taken over the last 2 weeks | ________ (in number) |  |
| 304 | Baseline CD4 cell count (from Card) | __________ (cell/µl) |  |
| 305 | Current CD4 cell count (from Card) | __________ (cell/µl) |  |
| 306 | Baseline viral load (from Card) | ____________ (No of copies/mL) |  |
| 307 | Current Viral load (from Card) | ____________ (No of copies/mL) |  |
| 308 | Baseline WHO clinical stage (from Card) | 1. Stage-I 2. Stage-II 3. Stage-III 4. Stage-IV |  |
| 309 | Current WHO clinical stage (from Card) | 1. Stage-I 2. Stage-II 3. Stage-III 4. Stage-IV |  |
| 310 | Presence of opportunistic infections (from Card) | 1. Yes 2. No | If “No” go-to Q312 |
| 311 | If “yes” to Q507 specify types of OI (s) | ______________________ |  |
| 312 | Current ART regimen (from Card) | 1. 1^st^ line 2. 2^nd^ line 3. 3^rd^ line |  |
| 313 | Duration since HIV diagnosis (from Card) | _______________ (in a year) |  |
| 314 | Current functional status (from Card) | 1. Working 2. Ambulatory 3. Bed ridden |  |
| 315 | Comorbidity (from Card) | 1. Yes 2. No |  |
| 316 | If comorbidity presented, specify it. |  |  |
| 317 | OI prophylaxis taken (from Card) | 1. Yes 2. No |  |
| 318 | Disclosure status (from Card) | 1. Disclosed 2. Not-disclosed |  |

**Part IV: Hospital Anxiety and Depression Scale (HADS)**

**Instruction:** Tick the box beside the reply that is closest to how you have been feeling in the past week. Don’t take too long over you replies: your immediate is best.

| **S. No** | **Questions** | **Respondents’ possible answers** |
| --- | --- | --- |
| **Anxiety measurement scale** | | |
| 401 | How frequently do you feel tense or 'wound up'? | 3. Most of the time 2. A lot of the time  1. From time to time, occasionally 0. Not at all |
| 402 | Do you get a sort of frightened feeling as if something awful is about to happen? | 3. Very definitely and quite badly 2. Yes, but not too badly  1. A little, but it doesn't worry me 0. Not at all |
| 403 | How frequently worrying thoughts go through my mind? | 3. A great deal of the time 2. A lot of the time  1. From time to time, but not often 0. Only occasionally |
| 404 | Can you sit at ease and feel relaxed? | 0. Definitely 1. Usually 2. Not Often 3. Not at all |
| 405 | How frequently do you get a sort of frightened feeling like 'butterflies' in the stomach? | 0. Not at all 1. Occasionally 2. Quite Often 3. Very often |
| 406 | Do you feel restless as you have to be on the move? | 3. Very much indeed 2. Quite a lot 1. Not very much  0. Not at all |
| 407 | How frequently do you get sudden feelings of panic? | 3. Very often indeed 2. Quite often 1. Not very often  0. Not at all |
| **Depression measurement scale** | | |
| 408 | Do you still enjoy the things you used to enjoy? | 0. Definitely as much 1. Not quite so much  2. Only a little 3. Hardly at all |
| 409 | Can you laugh and see the funny side of things? | 0. As much as I always could 1. Not quite so much now  2. Definitely not so much now 3. Not at all |
| 410 | How frequently do you feel cheerful? | 3. Not at all 2. Not often  1. Sometimes 0. Most of the time |
| 411 | How frequently do you feel as you are slowed down? | 3. Nearly all the time 2. Very often  1. Sometimes 0. Not at all |
| 412 | Have you lost interest in your appearance? | 3. Definitely  2. I don't take as much care as I should  1. I may not take quite as much care  0. I take just as much care as ever |
| 413 | Do you look forward with enjoyment to things? | 0. As much as I ever did 1. Rather less than I used to  2. Definitely less than I used to 3. Hardly at all |
| 414 | Can you enjoy a good book/radio/TV program? | 0. Often 1. Sometimes 2. Not often 3. Very seldom |

**Part 5: Questions related to social support.**

| S. No | Questions | | | Response | | | | | | |  |
| --- | --- | --- | --- | --- | --- | --- | --- | --- | --- | --- | --- |
| 501 | How many people are so close to you that you can count on them if you have serious problems? | | | 1. None 2. 1 or 2 3. 3 to5 4. 6 or more | | | | | | |  |
| 502 | How much concern do people show in what you are doing? | | | 1. No concern and interest 2. Little concern&interest   3. Uncertain 4. Some concern and interest   1. A lot of concern and interest | | | | | | |  |
| 503 | How easy can you get practical help from neighbors if you should need it? | | | 1. Very difficult 2. Difficult 3. Possible   4. Easy 5. Very easy | | | | | | |  |
| **Part 6: Questions related to perceived stigma**   \| S. No \|  \| **Options** \| \| \| \| \| \| --- \| --- \| --- \| --- \| --- \| --- \| --- \| \| Strongly disagree (1) \| Disagree (2) \| Neutral (3) \| Agree (4) \| Strongly agree (5) \| \| 601 \| Do you afraid about people discriminating you? \|  \|  \|  \|  \|  \| \| 602 \| are you worried that people may tell your HIV status to others? \|  \|  \|  \|  \|  \| \| 603 \| Did you tell people close to you to keep your HIV status as a secret? \|  \|  \|  \|  \|  \| \| 604 \| Do you think that people would be afraid of you if they knew your HIV status? \|  \|  \|  \|  \|  \| \| 605 \| Are you working hard to keep your HIV status as a secret? \|  \|  \|  \|  \|  \| \| 606 \| Do you feel that no one knows your HIV status? \|  \|  \|  \|  \|  \| \| 607 \| Do you feel guilty because of your HIV status? \|  \|  \|  \|  \|  \| \| 608 \| Do you feel you are a bad person due to your HIV status? \|  \|  \|  \|  \|  \| | | | | | | | | | |  |  |
| 609 | | Do people tell you that HIV is what you deserved? |  | |  |  |  |  |  | |  |
| 610 | | I feel I am unclean due to my HIV-positive status |  | |  |  |  |  |  | | |

**Part 7: Questions related to substance use**

| **S. No** | **Questions** | **Response** | **Skip** |
| --- | --- | --- | --- |
| 701 | In your life which of the following substances have you ever used (non-medical use only)? | - 1. Alcohol 2. Cigarette  1. Khat 4. Others (specify)______ | if none go-to 801 |
| 702 | In the past 3 months, have you used the substances you mentioned above? | - 1. Alcohol 2. Cigarette   3. Khat 4. Others (specify)____ |  |

**Part 8: questions on post-traumatic stress disorder**

| **S. No** | **Questions** | **Response** |
| --- | --- | --- |
| 801 | In the past month, have you had nightmares about the event(s) or thought about the event(s) when you did not want to? | 1. Yes 2. No |
| 802 | In the past month, have you tried hard not to think about the event(s) or went out of your way to avoid situations that reminded you of the event(s)? | 1. Yes 2. No |
| 803 | In the past month, have you been constantly on guard, watchful, or easily startled? | 1. Yes 2. No |
| 804 | In the past month, have you felt numb or detached from people, activities, or your surroundings? | 1. Yes 2. No |
| 805 | In the past month, have you felt guilty or unable to stop blaming yourself or others for the event(s) or any problems the event(s) may have caused? | 1. Yes 2. No |

**Part 9: Questions related to health-related quality of life (WHOQOL-HIV BREF)**

| **S/n** | **Questions** | **Very poor** | **Poor** | **Neither poor nor good** | **Good** | **Very good** |
| --- | --- | --- | --- | --- | --- | --- |
| 901 | How would you rate your quality of life? | 1 | 2 | 3 | 4 | 5 |
|  | | **Very dissatisfied** | **Dissatisfied** | **Neither**  **satisfied nor dissatisfied** | **Satisfied** | **Very satisfied** |
| 902 | How satisfied are you with your health? | 1 | 2 | 3 | 4 | 5 |
|  | | Not at all | A little | A moderate amount | Very much | An extreme amount |
| 903 | To what extent do you feel that physical pain prevents you from doing what you need to do? | 1 | 2 | 3 | 4 | 5 |
| 904 | How much are you bothered by any physical problems related to your HIV infection? | 1 | 2 | 3 | 4 | 5 |
| 905 | How much do you need any medical treatment to function in your daily life? | 1 | 2 | 3 | 4 | 5 |
| 906 | How much do you enjoy life? | 1 | 2 | 3 | 4 | 5 |
| 907 | To what extent do you feel your life to be meaningful? | 1 | 2 | 3 | 4 | 5 |
| 908 | To what extent are you bothered by people blaming you for your HIV status | 1 | 2 | 3 | 4 | 5 |
| 909 | How much do you fear the future? | 1 | 2 | 3 | 4 | 5 |
| 910 | How much do you worry about death? | 1 | 2 | 3 | 4 | 5 |
|  | | **Not at all** | **A little** | **A moderate amount** | **Very much** | **Extremely** |
| 911 | How well are you able to concentrate? | 1 | 2 | 3 | 4 | 5 |
| 912 | How safe do you feel in your daily life? | 1 | 2 | 3 | 4 | 5 |
| 913 | How healthy is your physical environment? | 1 | 2 | 3 | 4 | 5 |
|  | | **Not at all** | **A little** | **Moderately** | **Mostly** | **Completely** |
| 914 | Do you have enough energy for everyday life? | 1 | 2 | 3 | 4 | 5 |
| 915 | Are you able to accept your bodily appearance? | 1 | 2 | 3 | 4 | 5 |
| 916 | Have you enough money to meet your needs? | 1 | 2 | 3 | 4 | 5 |
| 917 | To what extent do you feel accepted by the people you know? | 1 | 2 | 3 | 4 | 5 |
| 918 | How available to you is the information that you need in your day-to-day life? | 1 | 2 | 3 | 4 | 5 |
| 919 | To what extent do you have the opportunity for leisure activities? | 1 | 2 | 3 | 4 | 5 |
|  | | **Very poor** | **Poor** | **Neither poor nor good** | **Good** | **Very good** |
| 920 | How well are you able to get around? | 1 | 2 | 3 | 4 | 5 |
|  | | **Very dissatisfied** | **Dissatisfied** | **Neither**  **satisfied nor dissatisfied** | **Satisfied** | **Very satisfied** |
| 921 | How satisfied are you with your sleep? | 1 | 2 | 3 | 4 | 5 |
| 922 | How satisfied are you with your ability to perform your daily living activities? | 1 | 2 | 3 | 4 | 5 |
| 923 | How satisfied are you with your capacity for work? | 1 | 2 | 3 | 4 | 5 |
| 924 | How satisfied are you with yourself? | 1 | 2 | 3 | 4 | 5 |
| 925 | How satisfied are you with your personal relationships? | 1 | 2 | 3 | 4 | 5 |
| 926 | How satisfied are you with your sex life? | 1 | 2 | 3 | 4 | 5 |
| 927 | How satisfied are you with the support you get from your friends? | 1 | 2 | 3 | 4 | 5 |
| 928 | How satisfied are you with the conditions of your living place? | 1 | 2 | 3 | 4 | 5 |
| 929 | How satisfied are you with your access to health services? | 1 | 2 | 3 | 4 | 5 |
| 930 | How satisfied are you with your transport? | 1 | 2 | 3 | 4 | 5 |
|  | | **Never** | **Seldom** | **Quite often** | **Very often** | **Always** |
| 931 | How often do you have negative feelings such as blue mood, despair, anxiety, depression? | 1 | 2 | 3 | 4 | 5 |

Thank you!

End.
